# Engraftment in NSG_SCF_ mice correlates with the WHO category and prognosis in systemic mastocytosis

**DOI:** 10.1038/s41375-023-01871-7

**Published:** 2023-03-14

**Authors:** Michael Willmann, Barbara Peter, Katharina Slavnitsch, Daniela Berger, Nadine Witzeneder, Gabriele Stefanzl, Gregor Eisenwort, Daniel Ivanov, Irina Sadovnik, Emir Hadzijusufovic, Georg Greiner, Tina Bernthaler, Gregor Hoermann, Maik Dahlhoff, Thomas Rülicke, Peter Valent

**Affiliations:** 1 Ludwig Boltzmann Institute for Hematology and Oncology, Medical University of Vienna, Vienna, Austria; 2 Department of Companion Animals, Clinic Unit of Internal Medicine, University of Veterinary Medicine Vienna, Vienna, Austria; 3 Institute of in-vivo and in-vitro Models, Department of Biomedical Sciences, University of Veterinary Medicine Vienna, Vienna, Austria; 4 Department of Medicine I, Clinical Division of Hematology and Hemostaseology, Medical University of Vienna, Vienna, Austria; 5 Munich Leukemia Laboratory (MLL), Munich, Germany

**Keywords:** Cancer stem cells, Cancer stem cells

**TO THE EDITOR:**

Systemic mastocytosis (SM) is a heterogeneous group of disorders characterized by abnormal growth and accumulation of mast cells (MC) in various organ-systems [1–3]. The classification of the World Health Organization (WHO) divides SM into non-advanced forms and advanced forms of the disease. Whereas patients with non-advanced SM, such as indolent SM (ISM) or smoldering SM (SSM) have an excellent prognosis, the prognosis and survival of patients with advanced SM, including aggressive SM (ASM), SM with an associated hematologic neoplasm (SM-AHN) and MC leukemia (MCL), are dismal [1–3].

Despite the prognostic value of the WHO classification, availability of additional prognostic variables, and recently developed prognostic scores, it is often difficult to predict the course and prognosis in individual patients [4, 5]. For example, even patients with ISM who have no or only a few risk factors concerning progression, may sometimes progress to ASM or MCL.

One strategy to define the expansion and progression of neoplastic cells may be to measure the dynamics of MC-related parameters such as the tryptase level or MC infiltration grade in the bone marrow (BM) [6, 7]. However, serum tryptase levels may also increase in ISM or SSM without signs of progression and serial BM investigations are usually not performed. It is also difficult to measure the proliferative capacity of neoplastic progenitors in patients with SM [8]. Another approach may be to measure the numbers and disease-propagating ability of neoplastic stem cells in patients with SM. However, the lack of suitable in vivo models reflecting non-advanced SM and advanced SM has limited previous attempts to correlate in vivo expansion of SM cells with clinical endpoints and prognosis.

The concept of leukemic stem cells (LSC) is well established in acute and chronic leukemias [9, 10]. More recently, we have identified LSC in patients with advanced SM, including MCL [11]. In most patients with MCL, LSC were found to produce detectable engraftment in NSG mice exhibiting membrane-bound human stem cell factor (NSG_SCF_) [11]. We also found that engraftment levels were low or minimal in those patients in whom a chronic (slowly expanding) form of MCL was diagnosed [11]. In order to demonstrate a definitive correlation between the WHO category of SM and the engraftment levels produced by neoplastic stem cells in NSG_SCF_ mice, we have extended our studies to a small series of patients with ISM and compared engraftment levels in ISM with engraftments detectable in advanced SM (ASM/MCL) in our previous study [11].

The experimental protocol applied was the same as the protocol used in our previous study on samples of patients with advanced SM [11]. Prior to BM investigations, patients gave written informed consent to participate. Aspirated BM cells were layered over Ficoll to isolate mononuclear cells (MNC), which were stored in a local biobank until used. The study was approved by the local ethics committee of the Medical University of Vienna (ECS 1184/2014 and 1063/2018). Xenotransplantation experiments were carried out as described [11]. In brief, 8–12 weeks old NOD-SCID-IL-2Rγ^−/−^ (NSG) mice expressing human membrane-bound SCF (NSG_SCF_ mice) purchased from The Jackson Laboratory (Bar Harbor, ME, USA; strain #: 017830) were sublethally irradiated (2.4 Gy) 24 h before injecting human cells prepared from 3 BM samples of patients with ISM (Table 1). Prior to injection, CD45^+^ and CD45^+^/CD34^─^ (stem cell-depleted) fractions of mononuclear BM cells were purified by flow cytometry-based cell sorting as described [11]. Isolated BM cells were resuspended in 0.15 ml RPMI medium containing 10% fetal calf serum. A total number of 1.6–2.5 × 10^5^ cells per mouse (5 mice in each cohort) were injected into the lateral tail vein of NSG_SCF_ mice. Animal studies were approved by the Ethics and Animal Welfare Committee of the University of Veterinary Medicine, Vienna in accordance with the University’s guidelines for Good Scientific Practice and authorized by the Austrian Federal Ministry of Education, Science and Research (BMWFW-68.205/0113-WF/V/3b/2016 and BMBWF-68.205/0221-V/3b/2019) in accordance with current legislation.

**Table 1:** Human cell engraftment in NSG_SCF_ mice injected with BM cells obtained from patients with ISM or advanced SM^a^.

#	Diagnosis	Injected cell fraction	Engraftment flow cytometry^b^	*TPSAB1*/*TPSB2* mRNA (qPCR)^c^	*KIT* (PCR)^d^
1	ISM	CD45^+^	0/5	0/5	0/5
		CD45^+^/CD34^−^	0/4	0/4	0/4
2	ISM	CD45^+^	0/5	3/5	2/5
		CD45^+^/CD34^−^	0/4	0/4	0/3
3	ISM	CD45^+^	0/5	5/5	5/5
		CD45^+^/CD34^−^	0/5	0/4	0/3
4	MCL	CD45^+^	3/3	n.t.	2/2
		CD45^+^/KIT^+^	3/3	n.t.	2/2
		CD45^+^/KIT^+^/CD34^−^	0/3	n.t.	2/2
5	MCL-AML	CD45^+^	3/3	2/3	3/3
		CD45^+^/KIT^+^	2/2	1/1	1/1
		CD45^+^/KIT^+^/CD34^-^	0/1	0/1	1/1
6	ASM-CMML	CD45^+^	3/4	1/1	2/2
		CD45^+^/KIT^+^	4/4	1/1	2/2
		CD45^+^/KIT^+^/CD34^−^	0/4	0/2	0/2
7	chronic MCL	CD45^+^	0/3	0/3	n.t.
		CD45^+^/KIT^+^	0/4	0/4	n.t.
		CD45^+^/KIT^+^/CD34^−^	0/4	0/4	n.t.
8	MCL	CD45^+^	4/4	2/2	2/2
		CD45^+^/KIT^+^	4/4	4/4	2/2
		CD45^+^/KIT^+^/CD34^−^	0/4	0/3	1/2
9	MCL-MDS/MPN	CD45^+^	0/4	3/4	n.t.
		CD45^+^/KIT^+^	0/4	2/4	n.t.
		CD45^+^/KIT^+^/CD34^−^	0/4	0/4	n.t.

Xenotransplantation experiments were performed essentially as described [11]. Bone marrow samples from 3 donors with ISM were stained using antibodies against CD34 and CD45, and total CD45^+^ cells as well as CD45^+^/CD34^─^ cells were purified by cell sorting on a FACSAria sorter (BD Biosciences). Then, cells were resuspended in RPMI medium containing 10% fetal calf serum and injected (1.6 × 10^5^–2.5 × 10^5^ cells per mouse, *n* = 5 mice per cohort and donor) into the tail vein of sublethally irradiated, 8–12 week-old, female NOD-SCID-IL-2Rγ^−/−^ (NOD.Cg-*Prkdc*^*scid*^ *Il2rg*^*tm1Wjl*^ Tg[PGK1-KITLG*220]441Daw/SzJ) mice exhibiting human SCF (NSG_SCF_). Mice were purchased from The Jackson Laboratory (number: JAX: 017830) and breeded as described [11]. Animals were inspected daily for occurrence of disease-related symptoms and sacrificed when such symptoms were seen or after a maximum period of 12 months. Mouse BM cells were obtained from flushed long bones and analyzed for engraftment of human cells by flow cytometry using antibodies against murine (mu)CD45, human (hu)CD45, huKIT, huCD19, huCD33, and DAPI (to exclude dead cells).

*ISM* indolent systemic mastocytosis, *ASM* aggressive systemic mastocytosis, *MCL* mast cell leukemia, *BM* bone marrow, *AML* acute myeloid leukemia, *MDS/MPN* myelodysplastic/myeloproliferative overlap neoplasm, *CMML* chronic myelomonocytic leukemia, *n.t.* not tested, *qPCR* quantitative real time PCR, *SCF* stem cell factor.

^a^Samples of cells of advanced SM patients were analyzed in a previous study [11].

^b^A cutoff of >0.5% huCD45^+^/huCD117^+^ for ISM mice or >0.5% of huCD117^+^ for ASM/MCL mice was used for determination of engraftment in the BM.

^c^A cutoff of >0.05% *TPSAB1*/*TPSB2* mRNA levels relative to mu-hu Actin mRNA levels in the BM of the mice was used for determination of *TPSAB1/TPSB2* (tryptase) mRNA expression levels. Actin was selected as control gene because of its cross-species (mu-hu) homology and cross-species abundance.

^d^Genomic *KIT* WT and *KIT* mutations were detected by ddPCR (droplet digital PCR) for ISM mice or by melting curve analysis after PCR clamping for ´ASM/MCL mice´.

After injection, mice were inspected daily and sacrificed when they showed disease symptoms or after a maximum observation time of 52 weeks. Then, mouse BM cells were obtained from flushed femurs, tibias, and humeri. Engraftment of human cells in the BM of NSG_SCF_ mice was quantified by flow cytometry using antibodies against murine (m)CD45, human (h)CD45, hKIT, and DAPI (to exclude dead cells). Engraftment was defined as the presence of at least 0.5% mCD45^─^/hCD45^+^/hKIT^+^ cells. Human MC engraftment was confirmed by qPCR or digital droplet PCR (ddPCR) using primers directed against human tryptase (*TPSAB1/TPSB2*) by qPCR or *KIT* by ddPCR.

As assessed by flow cytometry, no detectable engraftment with human cells was found in NSG_SCF_ mice injected with cells obtained from patients with ISM (Table 1). However, as assessed by immunohistochemistry, some human CD45^+^ cells engrafting the BM were detected in most mice examined (Fig. 1). Moreover, we were able to show that engrafting cells display human *KIT* and tryptase (*TPSAB1/TPSB2*) mRNA in our PCR experiments, suggesting that these cells were indeed MC or MC-committed progenitors (Table 1, Fig. 1). In aggregate, these data confirm that neoplastic stem cells in ISM can engraft in NSG_SCF_ mice, but engraftment levels assessed by flow cytometry are very low or not detectable in these patients. We also found that depletion of CD34^+^ stem cells resulted in a decrease of engraftment to undetectable level in all assays (Table 1).

**Fig. 1:**
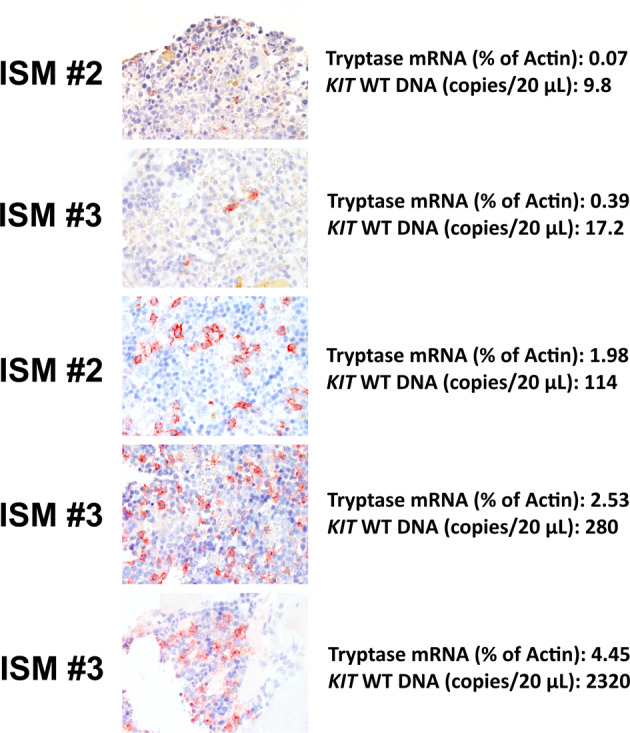
Detection of CD45^+^ human cells in the BM of NSG_SCF_ mice by immunohistochemistry staining and comparison with expression of tryptase mRNA and *KIT* DNA levels. Immunohistochemistry was performed on slides prepared from paraffin-embedded, formalin-fixed, pelvic bone specimens using the mouse on mouse (MOM) immunodetection kit (Vector Laboratories, Burlingham, CA, USA) according to the manufacturer’s protocol. A mouse monoclonal antibody against human CD45 (Dako, Glostrup, Denmark), diluted 1:50, was applied for 60 min at room temperature. MOM biotinylated anti-mouse IgG was used as secondary antibody. As peroxidase substrate, 3-amino-9-ethylcarabzole was applied. Slides were counterstained in Mayer´s hematoxylin. Pictures were taken at a magnification of 60 x. *TPSAB1/TPSB2* mRNA levels were measured by quantitative real-time PCR (qPCR) in BM cells flushed from long bones of NSG_SCF_ mice as follows: RNA was isolated using RNeasy Plus Mini Kit (Qiagen, Hilden, Germany). cDNA was synthesized using Moloney murine leukemia virus reverse transcriptase and random primers (Invitrogen, Carlsbad, CA). Primers for *TPSAB1/TPSB2* (forward: 5′- CGG GAA CAC CCG GAG GGA CT -3′and reverse: 5′ GCC TGC AGC CAG GTG CCA TT-3′) and *ACTB/Actb* (forward: 5′- TCG ACA ACG GCT CCG GCA TG-3′ and reverse: 5′- CCT CTC TTG CTC TGG GCC TCG TC-3′) were applied. qPCR was performed on a QuantStudio 3 Real-Time PCR System (Thermo Fisher Scientific, Waltham, MA, USA) using iTaq Universal SYBR Green Supermix (Bio-Rad, Hercules, CA, USA) and plasmid standards [11]. *TPSAB1*/*TPSB2* mRNA copy numbers were normalized to *ACTB*/*Actb* mRNA copy numbers and expressed as percent of *ACTB*/*Actb*. ´Detected expression´ of *TSPAB1*/*TPSB2* mRNA was defined by >0.05% *TSPAB1*/*TPSB2* mRNA relative to *ACTB*/*Actb* mRNA levels. To detect human *KIT* DNA in these samples, droplet digital PCR (ddPCR) was performed as follows: genomic DNA was extracted from FFPE pelvic mouse specimens using QIAamp DNA FFPE Tissue Kit (Qiagen, Hilden, DE). DNA concentrations were measured using a NanoDrop 1000 Spectrophotometer (Thermo Fisher Scientific, Waltham, MA). Approximately 280 ng of DNA per sample were used for ddPCR. DNA was pretreated with uracil DNA glycosylase (UDG, New England Biolabs, Ipswich, MA) for 20 min at 37 °C. ddPCR was performed with the Bio-Rad’s ddPCR Mutation Detection Assay (FAM + HEX) for human *KIT* wild-type. The reaction mixture additionally contained ddPCR Supermix for Probes (no dUTP, Bio-Rad, Hercules, CA) and HindIII-HF (New England Biolabs) restriction enzyme. The samples were fractionated into approximately 20000 droplets using a QX200 droplet generator. The PCR was then performed on a Gradient T100 Thermal Cycler (Bio-Rad) using the following protocol: 95 °C for 600 s (ramp rate 2 °C/second), 40 cycles at 94 °C for 30 s and 55 °C for 60 s (ramp rate 2 °C/second), 98 °C for 600 s and 4 °C (ramp rate 1 °C/second) until measurement. A QX200 droplet reader (Bio-Rad) was used for analysis.

So far, it remains unknown why no substantial engraftment of ISM-derived cells was detected by flow cytometry even in BM of mice where human MC were detected by immunohistochemistry and/or PCR. One explanation is that in ISM, most MC are tightly connected to their microenvironment resulting in low MC numbers in BM aspirates or flushed bones. By contrast, in advanced SM, MC are more frequently detected in aspirates and therefore may also be present in samples of flushed mouse BM [1, 12]. An alternative explanation may be that the numbers of engrafted cells in ISM were too low to be detected by flow cytometry. Indeed, it is well-known that immunohistochemistry and PCR may sometimes be more sensitive in comparison to flow cytometry. Finally, the numbers of neoplastic stem cells may be lower in patients with ISM compared to ASM and MCL.

Our data obtained in ISM patients are in contrast to the clearly demonstrable engraftment of neoplastic progenitor cells in NSG_SCF_ mice we detected in advanced SM in a previous study [11]. In particular, we found clearly measurable and reproducible engraftment in the BM with human cells when NSG_SCF_ mice were injected with cells obtained from patients with ASM and MCL (Table 1). It is worth noting that the mouse strain and methods employed to measure engraftment rates were largely the same in advanced SM and ISM. It is also noteworthy that even in patients with advanced SM, not all samples from all donors produced engraftment. For example, in one donor with chronic MCL, engraftment levels were low if at all detectable in NSG_SCF_ mice [11]. This observation is of particular importance as chronic MCL is considered a less aggressive variant of MCL where progression is only seen after several months even when the patient remains untreated [12].

All in all, our data suggest that the aggressiveness of SM correlates with the WHO type and with engraftment rates produced by neoplastic stem cells in NSG_SCF_ mice. Interestingly, even in WHO-defined subsets of SM, like MCL, the aggressiveness of SM may vary among patients, and the final outcome may depend on the ability of neoplastic stem cells to expand in vivo. Whether the biological behavior can indeed be predicted in all patients with SM and MCL by a xenotransplantation approach remains unknown. For example, several of these patients are also suffering from an AHN, and the AHN portion of the disease may or may not engraft in these mice [12]. In addition, MCL may develop as a leukemic or aleukemic variant and as a primary and secondary disease [12], and currently no data are available that would define the different engraftment levels and patterns in these patients. In several of the patients with non-advanced SM or chronic MCL, engraftment times may also take more than 6 or even 12 months. This prolonged engraftment has also been reported in a genetic mouse model employing *KIT* D816V [13]. In addition, the development of normal (non-neoplastic) MC from transplantable stem cells is considered to take several months [14].

So far, it remains unknown which factors and disease-related molecular drivers promote the oncogenic machineries and thus neoplastic stem cell expansion in vivo in SM. One factor may be the additional (KIT-independent) mutational landscape that has been described to correlate with disease progression and survival in SM [4, 15]. Unfortunately, we were not able to correlate mutation profiles with NSG_SCF_-engraftment rates in our patients with ISM or advanced SM because of the small number of cases examined and the fact that additional mutations are absent in almost all patients with ISM.

We conclude that neoplastic (stem) cells obtained from patients with ISM produce only minimal or no engraftment in NSG_SCF_ mice in flow cytometry experiments, contrasting the clearly detectable engraftment of neoplastic stem cells in patients with advanced SM.

## Data Availability

The datasets generated and/or analysed during the current study are available from the corresponding author on reasonable request.
